# Impact of Scheduled Outpatient Endoscopy Procedures on Inpatient Endoscopy Procedures: Prospective Analysis from a Single Tertiary Care Center

**DOI:** 10.1007/s10620-025-09322-2

**Published:** 2025-08-18

**Authors:** Ibrahim Yaghnam, Smriti Kochhar, Hadie Razjouyan

**Affiliations:** 1 Division of Gastroenterology and Hepatology, Penn State Health Milton S. Hershey Medical Center, Hershey, PA USA; 2 Division of Gastroenterology, University of Arizona, Banner Health, Tucson, AZ USA; 3 Division of Internal Medicine, Penn State Health Milton S. Hershey Medical Center, Hershey, PA USA

**Keywords:** Endoscopy scheduling, Inpatient endoscopy, Outpatient endoscopy, Weekend effect, Endoscopy delay

## Abstract

**Background and Study Aim:**

Over three million hospital admissions are made annually in the United States for gastrointestinal diseases, one-third of which result in inpatient endoscopic procedures. Delays in procedures have medical and financial implications, including prolonging hospital stays, decreasing quality measures, and reducing patient satisfaction. We aim to identify factors affecting inpatient procedures’ timely performance.

**Patients and Method:**

We conducted a prospective 15-week analysis of scheduled outpatient and inpatient procedures. We recorded the number of add-on procedures, including those completed in designated or alternate rooms, and the number of canceled and delayed procedures.

**Results:**

Of 1,467 total procedures, 473 were add-on inpatient procedures. Interventional procedures were significantly more likely to be delayed than general procedures (41.3 vs. 25.3%, *P* = 0.03). Delays were most frequent on Mondays (34%) and Tuesdays (30%). Mondays also had the highest mean number of add-on procedures, followed by Tuesdays (*P* < 0.01). Patients were most often returned without a procedure on Fridays (16.7%), followed by Mondays (13.3%) (*P* < 0.01).

Independent predictors of delayed procedures included the number of scheduled outpatient procedures (Odds Ratio [OR]: 6.76, 95% Confidence Interval [CI]: 2.24–20.35, *P*: < 0.01), number of added inpatient procedures (OR: 11.78, 95% CI: 4.82–28.82, *P*: < 0.01), and number of interventional cases (OR: 16.90, 95% CI: 11.76–24.26, *P*: < 0.01). Risk factors for patients being returned without a procedure included the number of add-ons (OR: 6.27, 95% CI: 2.16–18.17, *P* < 0.01) and scheduling on Fridays (OR: 9.74, 95% CI: 1.17–80.90, *P* = 0.03).

**Conclusion:**

High outpatient procedure volume on certain days impedes timely inpatient endoscopy. Adjusting schedules on high-burden days may reduce delays, improve access, and enhance outcomes.

**Supplementary Information:**

The online version contains supplementary material available at 10.1007/s10620-025-09322-2.

## Introduction

Endoscopy is essential for the diagnosis and treatment of various gastrointestinal conditions, and its use varies from diagnostic to therapeutic, including emergency medical treatments. The advancement and versatility of endoscopy has led to increasing demands, particularly in the inpatient setting. In the United States, over three million hospital admissions are due to gastrointestinal diseases, one-third of which require inpatient endoscopic procedures [1, 2]. Inpatient endoscopy delays can significantly impact clinical outcomes and healthcare resource utilization and are associated with prolonged hospital stays and increased readmission rates [3]. Inpatient procedures are impacted by outpatient scheduled procedures due to competition for endoscopy suite time and resources, leading to increased cancelation rates and delays.

Delays in completing inpatient endoscopic procedures add to the burden on the healthcare system, decreasing hospital-wide quality measures and patient satisfaction [2]. Inpatient endoscopy delays have both medical and financial implications [4].

Understanding outpatient endoscopy procedures’ impact on urgent and emergent inpatient endoscopies is critical for patient care, outcomes, and quality metrics. The primary aim of this study is to ascertain the factors that contribute to performing inpatient procedures in a timely manner in a unit accommodating outpatient procedures as well.

## Methods

This prospective observational study was conducted at the Pennsylvania State Milton S. Hershey Medical Center, a suburban tertiary care academic hospital over fifteen months, between June 2022 and August 2023. The endoscopy unit of the medical center has six active endoscopy rooms, all equipped for monitored anesthesia care with a dedicated anesthesia provider. Two of the six rooms have built-in fluoroscopy capability for interventional endoscopy. During a typical day, one fluoroscopy room is assigned to add-on inpatient interventional procedures, and one non-fluoroscopy room is dedicated for general add-on procedures. All the rooms have outpatient procedures scheduled throughout the day, with the exception of the inpatient-assigned rooms for general and interventional.

Depending on case complexity, the inpatient interventional room typically accommodates 2 to 3 inpatient procedures from 2:00 to 5:00 PM. In contrast, the inpatient general room offers 3 to 4 inpatient procedure slots—one around 11:30 AM to 12:00 PM and the remaining slots from 2:30 to 5:00 PM.

Endoscopy procedures are categorized into general endoscopy procedures or interventional endoscopy procedures. General endoscopy procedures include esophagogastroduodenoscopy (EGD), colonoscopy, or a combination of EGD and colonoscopy. For each EGD, a 30-min time slot is allocated. For a colonoscopy, a 40-min procedure time is allocated. Combined EGD and colonoscopy are allocated a 60-min time slot. Interventional procedures are performed with or without fluoroscopy, and include EGD, colonoscopy, endoscopic ultrasound (EUS), Endoscopic Retrograde Cholangiopancreatography (ERCP), combined EUS and ERCP, or device-assisted enteroscopy such as double balloon enteroscopy (DBE). We used a fixed time slot method during scheduling, where all interventional procedures are scheduled for 60 min except for DBE, which is allotted 90 to 120 min. Additionally, inpatient cases are scheduled for 30 min for general cases and 60 min for interventional cases. The time apportioned accounts for consent, transporting patients from the pre-procedure area, time out, and room turnover.

Each endoscopy room is staffed with a dedicated anesthesiology provider, a circulating nurse, and a technician. In addition to the gastroenterologist, there may be a fellow. Equipment and material are gathered by the circulating nurse, room cleaning and turnaround are performed by both the nurse and the technician, and patient transport to and from the procedure room is performed by the anesthesia provider.

### Data Collection

IY collected fifteen weeks’ worth of data, prospectively, over fifteen months. This was while IY was on gastroenterology consult service, which allowed for detailed data collection and analysis of inpatient add-ons and any reason for delays or cancelations. We reported the add-on procedures by patient-specific needs, meaning procedures were tallied regardless of the number of procedures needed. Each add-on can be for a single procedure (such as an EUS) or a double procedure (such as an EUS + ERCP). For outpatients, the therapeutic room has five morning outpatient slots, 60 min each with afternoon procedures scheduled in three 60-min slots from 2 to 5 pm. The general add-on room has six morning slots and six afternoon slots, varying from 30 min to 1 h.

The data was collected prospectively on a day-to-day basis. The number of add-on procedures was determined by 7:30 am. It was discussed during a morning huddle (Table 1). If an emergent procedure was placed on the add-on list, this procedure was included in the add-on tally. By 5 pm, the final number of add-ons requested, the number of procedures that were completed in their designated add-on room, the number of procedures that were completed in an alternate room (either an alternate endoscopy room, in the operating room, or at bedside), the number of canceled procedures, and the number of delayed procedures were tallied. All the patients were tallied at the end of the workday by IY.

**Table 1:**
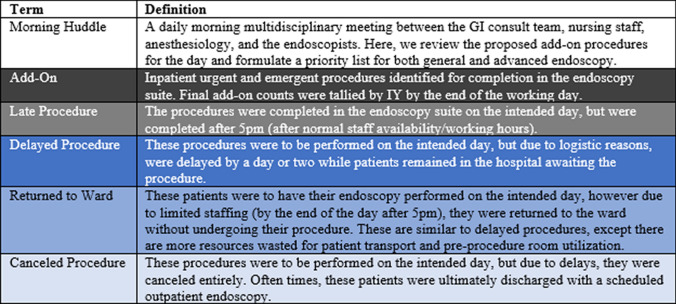
Table 1: Definitions and criteria used for case classification

### Definitions

Canceled procedures are those discussed in the morning huddle but were ultimately never completed, whereas delayed procedures were intended to be completed the same day but were completed in the following days—both due to logistic reasons (not including bowel preparation, oral intake, or anticoagulation use). Late procedures were emergent procedures completed in the endoscopy suite after 5 pm—which necessitated extending Anesthesia and nursing staffing hours beyond their scheduled times. Returned patients are those who were returned to the inpatient ward without a procedure after they arrived in the endoscopy suite with the intention of a procedure (Table 1).

### Statistical Analysis

Numerical variables are reported with mean + / − standard deviation. Univariate analysis was performed using a chi-squared test for categorical variables, a student’s *t*-test, and ANOVA where appropriate. Binary logistic regression analysis using backward stepwise elimination based on the probability of the Wald statistic was performed, including all clinical and statistically plausible variables. Nagelkerke *R*^2^ and the Hosmer–Lemeshow test were used to determine explanatory power and goodness of fit, respectively. A two-sided *P* value of < 0.05 was considered statistically significant. All variables that had a P value less than 0.2 in univariate analysis or had clinical plausibility were included in the regression model. Data analysis was conducted by utilizing the IBM Statistical Package for Social Sciences (IBM, Armonk, NY), version 29.

## Results

### Demographics

A total of 1,467 endoscopic procedures were completed in the two add-on procedure rooms over the course of the 15-week study. There were 890 general endoscopy procedures in the general add-on room, of which 648 were scheduled outpatient procedures and 242 were inpatient. A total of 577 interventional endoscopy procedures were performed in the interventional add-on room, of which 346 were scheduled outpatient procedures and 231 were inpatient add-on procedures.

**Fig. 1:**
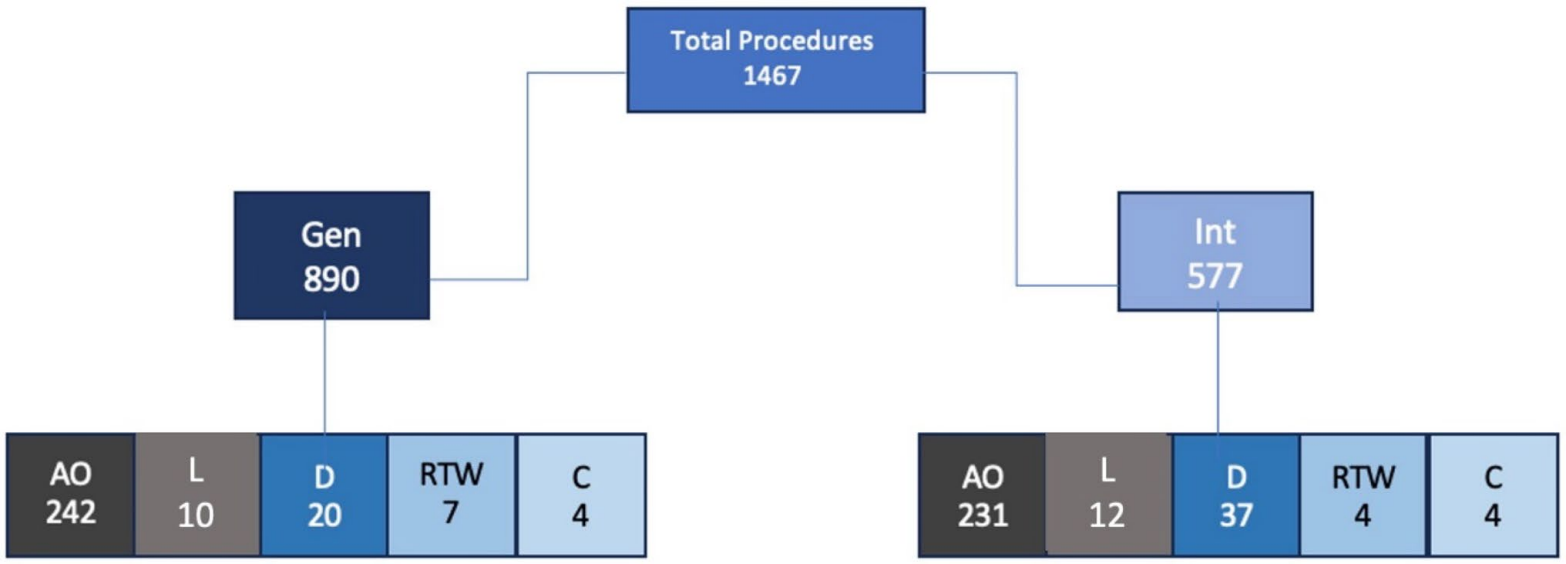
Breakdown of Total Procedures (*N* = 1467) into General Procedures (Gen) and Interventional Procedures (Int) with Categorization of Outcomes: *AO* Added On, *L* Late, *D* Delayed, *RTW* Return to Ward, and *C* Canceled

There were 994 cases of scheduled outpatient procedures in the two dedicated add-on rooms. 473 procedures were added as inpatient procedures. 20 procedures were completed after 5 pm (Late procedures). 57 procedures were delayed a day or two (Delayed procedures), and 10 patients returned to the ward as the procedure could not be done on the day of intended completion (Fig. 1) (Table 2).

**Table 2:**
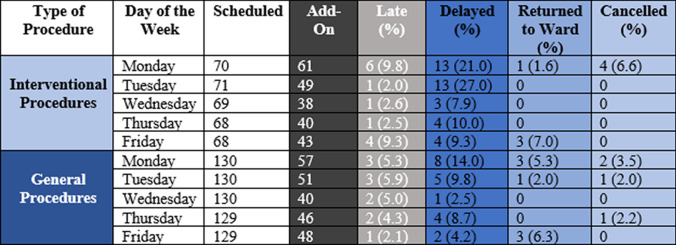
Procedural Outcomes by Day of Week and Type of Endoscopy

### Number of Procedure

There was no difference in the number of procedures added to either the general or interventional room (242 vs. 231, *P* = 0.50). There was no difference between general vs. interventional procedures being completed after hours (12 vs. 14.7%, *p* = 0.63) or patients returned (8 vs. 5.3%, *P* = 0.51). However, interventional procedures were more likely to be delayed by a day or more (41.3 vs. 25.3%, *p* = 0.03).

In binary logistic regression, the number of scheduled outpatient procedures (*P* < 0.01), the number of added inpatient procedures (*P* < 0.01), and interventional add-on procedures (*P* < 0.01) were independent predicting factors of delayed procedures (Table 3). The number of add-on inpatient procedures (OR: 6.27, 95% CI: 2.16–18.17, *P* < 0.01) was also an independent predictor for returning patients to the ward without a procedure.

**Table 3:**
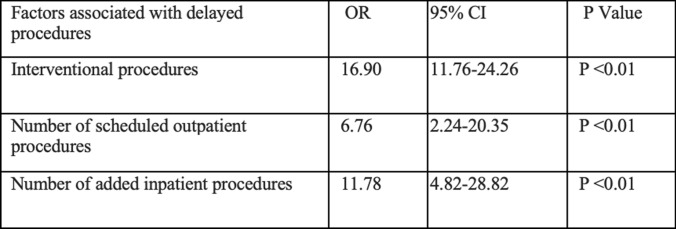
Factors associated with delayed procedures

The logistic regression model demonstrated strong explanatory power, with a Nagelkerke *R*^2^ of 0.57, and showed good fit to the observed data as indicated by the Hosmer–Lemeshow test (χ^2^ = 11.96, df = 8, *p* = 0.15), suggesting no significant lack of fit. The model also exhibited excellent discrimination ability, with an area under the receiver operating characteristic curve (AUC) of 0.883, indicating a high capability to distinguish cases with and without the delay accurately.

### Impact of Day of the Week

Friday had the statistically higher average number of add-on procedures, followed by Monday and Tuesday (*P* < 0.01). Patients were most frequently returned to the ward without a procedure on Friday, followed by Monday and then Thursday (*P* < 0.01) (Table 4). Delayed procedures were more common on Monday (34%) and Tuesday (30%).

**Table 4:**
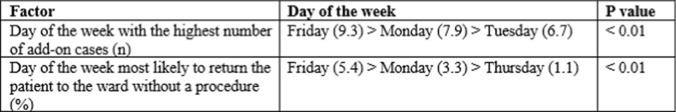
Table summarizing the impact of different days of the week on procedural outcomes

On binary logistic regression, scheduling a case on a Friday was an independent risk factor for returning a patient to the ward without a procedure (OR: 9.74, 95% CI: 1.17–80.90, *P* = 0.03). The logistic regression model showed moderate explanatory power (Nagelkerke *R*^2^ = 0.35) and a good fit (Hosmer–Lemeshow χ^2^ = 2.96, df = 8, *p* = 0.93). It demonstrated excellent discrimination, with an AUC of 0.858, indicating high accuracy in distinguishing patients who returned to the ward from those who did not.

## Discussion

This study identifies multiple logistic factors that influence the coordination of inpatient and outpatient procedures. The type of add-on procedure (general vs. therapeutic), the number of add-ons, and the days of the week play a role in procedure cancelation, delays, and likelihood of returning patients to the ward without a procedure.

### Type of Add-On Procedures

Interventional procedures were nearly 17 times more likely to be delayed compared to general procedures (41.3 vs. 25.3%, *p* 0.03). Interventional procedures are often more complex, technically demanding, and require specialized equipment and a multidisciplinary approach [5]. Furthermore, interventional procedures are sometimes performed on patients with surgically altered anatomy. This requires a thorough pre-operative evaluation to perform interventions safely and effectively [6]. It may require specialized techniques and precise navigation, which can significantly extend procedure time and may not always be accounted for during scheduling [7, 8]. In our study, patients were scheduled by pre-set time slots, which were created without accounting for altered anatomy or procedure complexity.

### Number of Add-On Procedures

The number of procedure add-ons also impacts the likelihood of delays. In our study, the probability of experiencing a delay increases by 6.76 and 11.78 times for each additional scheduled outpatient procedure and inpatient procedure, respectively. Preoperative steps such as obtaining informed consent and ensuring appropriate laboratory workup can be time-consuming [9]. Patients with multiple comorbidities may require even more complex scheduling and coordination with multidisciplinary teams. It is recommended that these patients would benefit from a multidisciplinary care coordination team to prevent prolonged length of stay [10, 11].

To ensure the smooth functioning of an endoscopic unit, dedicated endoscopy personnel and the necessary unit resources are essential. Lack of endoscopy personnel and unit availability accounted for 24.4% of inpatient endoscopy delays. Furthermore, the efficiency of the endoscopy suite itself plays a role. Though not noted as part of this study, delays can occur due to inefficiencies of the endoscopy unit, including a slow pre-op process and delays in room turnover time. These delays are associated with prolonged hospital length of stay and increased 30-day readmission rates, underscoring the impact of resource limitations on patient outcomes. These operational inefficiencies may also lead to prolonged fasting status, increased patient anxiety, and extended hospital length of stay, particularly for inpatients awaiting endoscopy [3, 12].

### Days of the Week Impact on Schedules

Delays in add-on inpatient procedures are associated with the scheduled day of the week. The highest rates of procedure delays occurred on Mondays (34%), followed by Tuesdays (30%). High hospital occupancy at the beginning of the week and an accumulation of patients over the weekend often contribute to delays, with Mondays and Tuesdays generating nearly 42% of scheduled patient occupancy time for non-ICU patients [13]. This may be partly due to a backlog of cases from the weekend as many non-urgent procedures are not performed [14]. This backlog can overwhelm available resources, cause delays, and even return patients back to the ward without a procedure, as seen in our study, with 13.3% of patients returning to the ward without a procedure on Monday. Additionally, variability in outpatient settings, patient attendance, and compliance affect scheduling efficiency, with the highest rates of non-attendance on Mondays [15]. The “weekend effect” has also been reported, and it refers to the observation that patients admitted to hospitals on Friday or the weekends may experience worse outcomes compared to those admitted on weekdays, potentially due to reduced staffing and resource availability [16].

Fridays have been reported to be associated with a higher number of gastrointestinal consults, which can overwhelm available resources and lead to delays in procedure scheduling. Interventional procedures are more likely to get delayed on Fridays due to several factors, including reduced staffing levels and the accumulation of cases from earlier in the week [17]. All the above factors can be the cause of patients being 10 times more likely to be returned to the ward without a procedure on Friday.

### Limitations

The limitations of this study include the fact that it was done over 15 weeks at a single tertiary care center, which may limit the generalizability of the findings. Additionally, being an academic center, there are fellows at varying levels of training, which could have influenced both procedure efficiency and outcomes. For example, it was previously reported that fellow involvement prolonged the procedure time by 10–37%, depending on the type of endoscopy performed [18]. Additional studies confirm that trainee participation did add to procedure duration [19, 20].

There are several reasons resulting in patients returning to the ward without a procedure, as it can be due to medical complexity, not qualifying for anesthesia, or inability to add patients to the schedule due to loss in staffing at the end of the day. Interestingly, logistic inpatient factors causing delays were previously reported to be avoidable. These factors include nursing staff availability, anesthesia availability, and the volume of scheduled outpatient procedures [21]. A multidisciplinary approach, including extending working hours and reducing outpatient procedure volumes, was suggested to improve inpatient access to endoscopy. In our study, we focused on the scheduled outpatient procedure volume as a direct competitor to inpatient add-ons. Staffing, including nursing and anesthesia availability, was inherently included in our analysis as they are the limitation to the 5 pm cut-off for procedures. Our study supports the previously reported findings and further elaborates on the day of the week and the type of procedure as factors not previously reported on.

Other factors not accounted for in the study include patient-related issues such as late arrivals for outpatient procedures, delays in patient transport for inpatient procedures, pre-procedural preparation (pre-op assessment and IV access), and non-attendance (no show patients for outpatient procedures). Same-day surgical procedures can be burdensome to patients who need to arrange their transport, causing them to reschedule or make alternative arrangements [20, 22].

There is limited prospective data published on the workflow of endoscopic units at tertiary care centers, making direct comparisons difficult. Previously, a retrospective analysis demonstrated factors associated with inpatient endoscopy delay [3]. These factors were poor bowel preparation and a lack of endoscopy personnel or unit availability. The latter problem is a logistic concern, which we elaborate on in this study. We believe the benefit of this prospective design is that it allows us to elucidate the logistic concerns by type of procedure, number of add-ons, and the day of the week. This detailed analysis is further illustrated by having a single person (IY) perform data collection while on the inpatient GI service, such that all detailed procedure logistics were discussed with him in real time. Bowel preparation was not included in our analysis, as patients who did not complete their bowel preparation were not included in the add-on list, which was finalized by the end of the day.

Lastly, an important benchmark in endoscopy suite efficiency is procedure room turnover time, which is defined as the crucial time between procedures during which cleaning and preparing the room takes place. The turnover time is not included in this study, and this can certainly be an important factor in procedural efficiency and completing inpatient procedures. Also, we do not report on urgent versus emergent indications for procedures. We acknowledge this is an important factor in determining which procedures are completed versus delayed, and this may bias some of our results. Generally, however, in the morning huddle, we prioritize the emergent procedures to ensure these are completed first, then urgent procedures followed by non-urgent procedures.

## Conclusion

In summary, the number of add-on inpatient procedures, the type of add-on procedures, and specific days of the week were identified as the primary factors influencing the timely completion of inpatient endoscopic procedures, while addressing a literature gap on operational workflows.

The number of inpatient add-on procedures and the type of add-on procedure are factors that are beyond the control of intervention. However, addressing the allocation of endoscopy time for inpatient procedures on high-access add-on days can help mitigate the burden of inpatient add-on procedures. Specifically, increasing the availability of inpatient procedure slots at the beginning and end of the week may alleviate scheduling challenges.

The interplay between outpatient and inpatient scheduling is complex, with delays influenced not only by procedure volume but also by the day of the week. To improve operational efficiency, reducing outpatient procedure slots on Mondays, Tuesdays, and Fridays or increasing staffing during these high-demand days could help minimize inpatient delays and the need for patient returns to the wards.

Additionally, further studies with a focus on analyzing pre-op preparation and procedure room turnover time could identify additional factors contributing to procedural delays, particularly on high-access days like Mondays and Fridays.

Future implementation and systematic evaluation of these strategies could optimize resource utilization and enhance procedural timeliness in tertiary care centers, ultimately improving patient outcomes.

## Supplementary Information

Below is the link to the electronic supplementary material.

Supplementary file1 (DOCX 37 KB)

## Data Availability

Data available upon request from the corresponding author, IY.

## Abbreviation

EUS: Endoscopic ultrasound
ERCP: Endoscopic retrograde cholangiopancreatography
EGD: Esophagogastroduodenoscopy
DBE: Double balloon enteroscopy
