# Supplementary material for: Impact of Scheduled Outpatient Endoscopy Procedures on Inpatient Endoscopy Procedures: Prospective Analysis from a Single Tertiary Care Center
Source: Dig Dis Sci. 2025 Aug 18;71(1):121–8. doi: 10.1007/s10620-025-09322-2 (PMC12909401; doi:10.1007/s10620-025-09322-2)
Supplement: Supplementary file 1 — Supplementary file1 (DOCX 37 KB) [file 10620_2025_9322_MOESM1_ESM.docx]

**Logistic Regression**

| **Notes** | | |
| --- | --- | --- |
| Output Created | | 05-JUL-2025 09:41:36 |
| Comments | |  |
| Input | Data | C:\Users\hadir\Desktop\USB 4_5_2025\QI project\Untitled2.sav |
|  | Active Dataset | DataSet3 |
|  | Filter | <none> |
|  | Weight | <none> |
|  | Split File | <none> |
|  | N of Rows in Working Data File | 150 |
| Missing Value Handling | Definition of Missing | User-defined missing values are treated as missing |
| Syntax | | LOGISTIC REGRESSION VARIABLES Dl_0_1 /METHOD=BSTEP(WALD) Sc Ao G0_T1 /SAVE=PRED COOK LEVER DFBETA /CASEWISE OUTLIER(2) /PRINT=GOODFIT CORR ITER(1) CI(95) /CRITERIA=PIN(0.05) POUT(0.10) ITERATE(20) CUT(0.5). |
| Resources | Processor Time | 00:00:00.02 |
|  | Elapsed Time | 00:00:00.02 |
| Variables Created or Modified | PRE_1 | Predicted probability |
|  | COO_1 | Analog of Cook's influence statistics |
|  | LEV_1 | Leverage value |
|  | DFB0_1 | DFBETA for constant |
|  | DFB1_1 | DFBETA for Sc |
|  | DFB2_1 | DFBETA for Ao |
|  | DFB3_1 | DFBETA for G0_T1 |

| **Case Processing Summary** | | | |
| --- | --- | --- | --- |
| Unweighted Cases^a^ | | N | Percent |
| Selected Cases | Included in Analysis | 150 | 100.0 |
|  | Missing Cases | 0 | .0 |
|  | Total | 150 | 100.0 |
| Unselected Cases | | 0 | .0 |
| Total | | 150 | 100.0 |
| a. If weight is in effect, see classification table for the total number of cases. | | | |

| **Dependent Variable Encoding** | |
| --- | --- |
| Original Value | Internal Value |
| .00 | 0 |
| 1.00 | 1 |

Block 0: Beginning Block

| **Iteration History**^a,b,c^ | | | |
| --- | --- | --- | --- |
| Iteration | | -2 Log likelihood | Coefficients |
|  |  |  | Constant |
| Step 0 | 1 | 190.978 | -.667 |
|  | 2 | 190.954 | -.693 |
|  | 3 | 190.954 | -.693 |
| a. Constant is included in the model. | | | |
| b. Initial -2 Log Likelihood: 190.954 | | | |
| c. Estimation terminated at iteration number 3 because parameter estimates changed by less than .001. | | | |

| **Classification Table**^a,b^ | | | | | |
| --- | --- | --- | --- | --- | --- |
|  | Observed | | Predicted | | |
|  |  |  | Dl_0_1 | | Percentage Correct |
|  |  |  | .00 | 1.00 |  |
| Step 0 | Dl_0_1 | .00 | 100 | 0 | 100.0 |
|  |  | 1.00 | 50 | 0 | .0 |
|  | Overall Percentage | |  |  | 66.7 |
| a. Constant is included in the model. | | | | | |
| b. The cut value is .500 | | | | | |

| **Variables in the Equation** | | | | | | | |
| --- | --- | --- | --- | --- | --- | --- | --- |
|  | | B | S.E. | Wald | df | Sig. | Exp(B) |
| Step 0 | Constant | -.693 | .173 | 16.015 | 1 | <.001 | .500 |

| **Variables not in the Equation** | | | | | |
| --- | --- | --- | --- | --- | --- |
|  | | | Score | df | Sig. |
| Step 0 | Variables | Sc | 2.330 | 1 | .127 |
|  |  | Ao | 43.813 | 1 | <.001 |
|  |  | G0_T1 | 4.320 | 1 | .038 |
|  | Overall Statistics | | 55.505 | 3 | <.001 |

Block 1: Method = Backward Stepwise (Wald)

| **Iteration History**^a,b,c,d^ | | | | | | |
| --- | --- | --- | --- | --- | --- | --- |
| Iteration | | -2 Log likelihood | Coefficients | | | |
|  |  |  | Constant | Sc | Ao | G0_T1 |
| Step 1 | 1 | 131.246 | -10.011 | .677 | .984 | 3.510 |
|  | 2 | 115.722 | -17.882 | 1.252 | 1.694 | 6.439 |
|  | 3 | 112.095 | -24.038 | 1.711 | 2.234 | 8.739 |
|  | 4 | 111.795 | -26.455 | 1.892 | 2.444 | 9.637 |
|  | 5 | 111.792 | -26.717 | 1.912 | 2.467 | 9.734 |
|  | 6 | 111.792 | -26.720 | 1.912 | 2.467 | 9.735 |
|  | 7 | 111.792 | -26.720 | 1.912 | 2.467 | 9.735 |
| a. Method: Backward Stepwise (Wald) | | | | | | |
| b. Constant is included in the model. | | | | | | |
| c. Initial -2 Log Likelihood: 190.954 | | | | | | |
| d. Estimation terminated at iteration number 7 because parameter estimates changed by less than .001. | | | | | | |

| **Omnibus Tests of Model Coefficients** | | | | |
| --- | --- | --- | --- | --- |
|  | | Chi-square | df | Sig. |
| Step 1 | Step | 79.162 | 3 | <.001 |
|  | Block | 79.162 | 3 | <.001 |
|  | Model | 79.162 | 3 | <.001 |

| **Model Summary** | | | |
| --- | --- | --- | --- |
| Step | -2 Log likelihood | Cox & Snell R Square | Nagelkerke R Square |
| 1 | 111.792^a^ | .410 | .570 |
| a. Estimation terminated at iteration number 7 because parameter estimates changed by less than .001. | | | |

| **Hosmer and Lemeshow Test** | | | |
| --- | --- | --- | --- |
| Step | Chi-square | df | Sig. |
| 1 | 11.964 | 8 | .153 |

| **Contingency Table for Hosmer and Lemeshow Test** | | | | | | |
| --- | --- | --- | --- | --- | --- | --- |
|  | | Dl_0_1 = .00 | | Dl_0_1 = 1.00 | | Total |
|  |  | Observed | Expected | Observed | Expected |  |
| Step 1 | 1 | 15 | 14.965 | 0 | .035 | 15 |
|  | 2 | 19 | 18.742 | 0 | .258 | 19 |
|  | 3 | 11 | 10.171 | 0 | .829 | 11 |
|  | 4 | 13 | 15.166 | 4 | 1.834 | 17 |
|  | 5 | 12 | 11.362 | 1 | 1.638 | 13 |
|  | 6 | 6 | 7.158 | 3 | 1.842 | 9 |
|  | 7 | 10 | 11.139 | 12 | 10.861 | 22 |
|  | 8 | 9 | 4.949 | 3 | 7.051 | 12 |
|  | 9 | 5 | 5.679 | 11 | 10.321 | 16 |
|  | 10 | 0 | .667 | 16 | 15.333 | 16 |

| **Classification Table**^a^ | | | | | |
| --- | --- | --- | --- | --- | --- |
|  | Observed | | Predicted | | |
|  |  |  | Dl_0_1 | | Percentage Correct |
|  |  |  | .00 | 1.00 |  |
| Step 1 | Dl_0_1 | .00 | 86 | 14 | 86.0 |
|  |  | 1.00 | 20 | 30 | 60.0 |
|  | Overall Percentage | |  |  | 77.3 |
| a. The cut value is .500 | | | | | |

| **Variables in the Equation** | | | | | | | | |
| --- | --- | --- | --- | --- | --- | --- | --- | --- |
|  | | B | S.E. | Wald | df | Sig. | Exp(B) | 95% C.I.for EXP(B) |
|  |  |  |  |  |  |  |  | Lower |
| Step 1^a^ | Sc | 1.912 | .562 | 11.573 | 1 | <.001 | 6.765 | 2.249 |
|  | Ao | 2.467 | .456 | 29.233 | 1 | <.001 | 11.785 | 4.819 |
|  | G0_T1 | 9.735 | 2.534 | 14.756 | 1 | <.001 | 16.896 | 11.765 |
|  | Constant | -26.720 | 6.069 | 19.382 | 1 | <.001 | .000 |  |

| **Variables in the Equation** | | | |  |  |  |  |  |  |
| --- | --- | --- | --- | --- | --- | --- | --- | --- | --- |
|  | | 95% C.I.for EXP(B) | |  |  |  |  |  |  |
|  |  | Upper | |  |  |  |  |  |  |
| Step 1^a^ | Sc | 20.354 | |  |  |  |  |  |  |
|  | Ao | 28.820 | |  |  |  |  |  |  |
|  | G0_T1 | 24.261 | |  |  |  |  |  |  |
|  | Constant |  | |  |  |  |  |  |  |
|  |  |  |  | |  |  |  |  |  |

| a. Variable(s) entered on step 1: Sc, Ao, G0_T1. |
| --- |

| **Correlation Matrix** | | | | | |
| --- | --- | --- | --- | --- | --- |
|  | | Constant | Sc | Ao | G0_T1 |
| Step 1 | Constant | 1.000 | -.970 | -.735 | -.977 |
|  | Sc | -.970 | 1.000 | .555 | .978 |
|  | Ao | -.735 | .555 | 1.000 | .610 |
|  | G0_T1 | -.977 | .978 | .610 | 1.000 |

| **Casewise List**^b^ | | | | | | | |
| --- | --- | --- | --- | --- | --- | --- | --- |
| Case | Selected Status^a^ | Observed | Predicted | Predicted Group | Temporary Variable | | |
|  |  | Dl_0_1 |  |  | Resid | ZResid | SResid |
| 48 | S | 1** | .108 | 0 | .892 | 2.876 | 2.135 |
| 51 | S | 1** | .108 | 0 | .892 | 2.876 | 2.135 |
| 53 | S | 1** | .108 | 0 | .892 | 2.876 | 2.135 |
| 56 | S | 1** | .108 | 0 | .892 | 2.876 | 2.135 |
| 143 | S | 1** | .126 | 0 | .874 | 2.634 | 2.065 |
| a. S = Selected, U = Unselected cases, and ** = Misclassified cases. | | | | | | | |
| b. Cases with studentized residuals greater than 2.000 are listed. | | | | | | | |

**Paragraph**

**ROC Analysis**

| **Notes** | | |
| --- | --- | --- |
| Output Created | | 05-JUL-2025 09:47:30 |
| Comments | |  |
| Input | Data | C:\Users\hadir\Desktop\USB 4_5_2025\QI project\Untitled2.sav |
|  | Active Dataset | DataSet3 |
|  | Filter | <none> |
|  | Weight | <none> |
|  | Split File | <none> |
|  | N of Rows in Working Data File | 150 |
| Missing Value Handling | Definition of Missing | User-defined missing values are treated as missing. |
|  | Cases Used | Only cases with valid data for all analysis variables are used in computing any statistics. |
| Weight Handling | | not applicable |
| Syntax | | ROC ANALYSIS PRE_1 BY Dl_0_1 (1) /MISSING USERMISSING=EXCLUDE /CRITERIA CUTOFF=INCLUDE TESTPOS=LARGE DISTRIBUTION=FREE CI=95 /DESIGN PAIR=FALSE /PLOT CURVE=ROC MODELQUALITY=TRUE /PRINT SE=FALSE CLASSIFIER=FALSE. |
| Resources | Processor Time | 00:00:04.02 |
|  | Elapsed Time | 00:00:01.09 |

| **Case Processing Summary** | |
| --- | --- |
| Dl_0_1 | Valid N (listwise) |
| Positive^a^ | 50 |
| Negative | 100 |
| Missing | 0 |
| Total | 150 |
| Larger values of the test result variable(s) indicate stronger evidence for a positive actual state. | |
| a. The positive actual state is 1.00. | |

| **Area Under the ROC Curve** |
| --- |
| Test Result Variable(s): Predicted probability |
| Area |
| .883 |
| The test result variable(s): Predicted probability has at least one tie between the positive actual state group and the negative actual state group. Statistics may be biased. |
